# The Effect of Artificial Sweeteners Use on Sweet Taste Perception and Weight Loss Efficacy: A Review

**DOI:** 10.3390/nu14061261

**Published:** 2022-03-16

**Authors:** Klara Wilk, Wiktoria Korytek, Marta Pelczyńska, Małgorzata Moszak, Paweł Bogdański

**Affiliations:** 1 Student Scientific Club of Clinical Dietetics, Department of the Treatment of Obesity and Metabolic Disorders and Clinical Dietetics, Poznań University of Medical Sciences, 60-569 Poznań, Poland; klara.wilk1998@gmail.com (K.W.); wiktoriakorytek@gmail.com (W.K.); 2 Department of Treatment of Obesity, Metabolic Disorders and Clinical Dietetics, Poznań University of Medical Sciences, 60-569 Poznań, Poland; mpelczynska@ump.edu.pl (M.P.); pbogdanski@ump.edu.pl (P.B.)

**Keywords:** sweet taste, non-nutritive sweeteners, body weight, obesity

## Abstract

Excessive consumption of sugar-rich foods is currently one of the most important factors that has led to the development of the global pandemic of obesity. On the other hand, there is evidence that obesity contributes to reduced sensitivity to sweet taste and hormonal changes affecting appetite, leading to an increased craving for sweets. A high intake of sugars increases the caloric value of the diet and, consequently, leads to weight gain. Moreover, attention is drawn to the concept of the addictive properties of sugar and sugary foods. A potential method to reduce the energy value of diet while maintaining the sweet taste is using non-nutritive sweeteners (NNS). NNS are commonly used as table sugar substitutes. This wide group of chemical compounds features high sweetness almost without calories due to its high sweetening strength. NNS include aspartame, acesulfame-K, sucralose, saccharin, cyclamate, neohesperidin dihydrochalcone (neohesperidin DC), neotame, taumatin, and advantame. The available evidence suggests that replacing sugar with NNS may support weight control. However, the effect of NNS on the regulation of appetite and sweet taste perception is not clear. Therefore, the review aimed to summarize the current knowledge about the use of NNS as a potential strategy for weight loss and their impact on sweet taste perception. Most studies have demonstrated that consumption of NNS-sweetened foods does not increase sweetness preference orenergy intake. Nonetheless, further research is required to determine the long-term effects of NNS on weight management.

## 1. Introduction

Excessive consumption of sugar-rich foods is one of the most important factors leading to the global pandemic of obesity. The prevalence of overweight and obesity is estimated to have almost tripled over the past five decades. The number of overweight individuals reached over 1.9 billion in 2016 [1]. According to the World Health Organization (WHO), daily energy intake from added sugars should not exceed 5–10% [2]. Unfortunately, statistics indicate that, in many countries, sugar consumption is significantly higher [3].

Taste influences eating behavior and the desire to eat certain products, which is related to the total of energy provided to the body with food. High sugar content in the diet can increase the risk of developing obesity [4]. Differences in taste function observed in obese vs. lean individuals may have an impact on the amount of consumed sugar and can lead to energy imbalance [5]. A potential strategy of reducing the global obesity problem is limiting sugar supply through its alternatives. For this purpose, non-nutritive sweeteners are commonly used in the food industry. These chemical compounds are several hundred to several thousand times sweeter than sucrose. Most NNS approved for use are derived synthetically. Due to their intense sweetening power, NNS used in small amounts can significantly reduce the energy value of products and simultaneously maintain their palatability [6]. NNS-sweetened foods enjoy great interest, and their consumption across all age groups is steadily growing [7]. Based on data from the NHANES (National Health and Nutrition Examination Survey) study conducted in 2007–2012, nearly 50% of American adults report regular consumption of foods containing NNS, particularly sugar-free beverages [8].

The replacement of sugar with NNS as a weight control strategy is still controversial [9]. Although the use of NNS under certain conditionsis considered safe for the general population, little is known about the long-term health effects [10]. In the majority of cases, studies only aim at short-term effects of NNS. Longitudinal results are still missing to draw conclusions for the future [10].

Due to physiological differences in the perception of sweet taste after consumption of NNS and their diverse effect on hunger and satiety centers, it has been assumed that the ingestion of foods containing NNS may not be an effective method to reduce energy intake for weight loss. Moreover, it has been suggested that frequent exposure to NNS may even increase appetite for sweet foods [8]. This fact seems to be an important goal in treating obesity because an inverse correlation between body mass index (BMI) and sweet taste sensitivity has been observed among overweight individuals [11,12,13]. For example, a study by Proserpio et al. found that obese individuals have lower sensitivity to all primary tastes than normal-weight individuals [14]. A lower sweetness detection threshold may lead to greater sugar- and carbohydrate-rich foods consumption (to achieve the desired sweet taste intensity). Nevertheless, other studies do not support the relationship between excessive body weight and increased craving for sweet foods [15,16].

This review aims to summarize the current knowledge on the use of NNS as a potential strategy for obesity treatment and their impact on the perception of sweet taste and the efficacy of weight loss.

## 2. Artificial Sweeteners-Definition, Types, Characteristics

NNS are commonly used as table sugar replacements. This wide group of chemical compounds is characterized by high sweetening intensity and low energy value. They are classified as food additives and considered by the European Food Safety Authority (EFSA) to be safe for use in doses not exceeding the acceptable daily intake (ADI). The following NNS have been approved for use in the European Union: aspartame, acesulfame-K, sucralose, cyclamate, neohesperidin DC, thaumatin, neotame, advantame, saccharin, and aspartame-acesulfame salt [17].

In the United States, the Food and Drug Administration (FDA) has approved eight NNS for use, including six derived synthetically (aspartame, acesulfame-K, neotame, sucralose, saccharin, and advantame) and two of natural origin (stevia, monk fruit extract/Luo Han Guo) [18] (Table 1).

## 3. Sweet Taste Perception

### 3.1. Physiological and Neurobiological Mechanism of Sweet Taste Perception

Sweet taste is one of the five flavors identified by human taste buds, which consist of G protein-coupled receptor cells (TRC) [13]. Different types of taste receptors are responsible for the perception of various taste stimuli, and the perception of sweetness is conditioned by the activation of the type 1 receptor (T1R), composed of T1R1, T1R2, and T1R3 subunits. However, only T1R2 and T1R3 subunits are involved in this process, whereas T1R1 plays a role in umami taste detection [13]. Sweet taste receptors are located in the oral cavity and gastrointestinal epithelial cells, pancreatic islets, adipose tissue, respiratory, and genitourinary structures [13]. The binding of sweet-tasting molecules to the T1R subunit, i.e., both simple and natural sugars, artificial sweeteners, as well as some amino acids and proteins, results in stimulation of the G protein. The cascade of reactions causes the stimulation of the appropriate receptors located on afferent nerve fibers that mediate the taste signal from T1R2 and T1R3 to brain centers, including the hypothalamus [13].

The enteroendocrine cells of the gastrointestinal tract, mainly L and K cells, also participate in the sweet taste signal transmission. As a result of stimulation of an appropriate T1R subunit, they secrete bioactive peptides, which have both local and peripheral effects on other tissues and organs and the central nervous system. Among them, there are incretin hormones (glucagon-like peptide-1–GLP-1, gastric inhibitory peptide—GIP) and a neurotransmitter—serotonin (5-HT). These compounds stimulate insulin secretion in response to glucose ingestion and signal satiety, thereby regulating the body’s energy metabolism [13]. Additionally, peptides secreted by taste cells and gastrointestinal enteroendocrine cells, including leptin, ghrelin, peptide YY, and cholecystokinin, modulate sensory stimuli [19].

The complex of neurons located in the arcuate nucleus (ARC) of the hypothalamus constitutes the center regulating energy homeostasis. These neuronal systems possess receptors for specific hormones regulating hunger and satiety and can detect energy substrates. The most important role in this process is played by the orexigenic system (stimulating appetite), formed by neuropeptide Y (NPY) and the Agouti-related protein (AgRP), as well as the anorexigenic system (inhibiting food intake), constituting a complex of proopiomelanocortin neurons (POMC) [15]. Increased secretion of GLP-1 and GIP due to stimulation of sweet taste receptors is a signal to stop food intake, received and processed by both systems. Subsequently, it is transmitted to other centers of the hypothalamus: the paraventricular nucleus (PVC) and the hunger center–lateral hypothalamus (LHA). Finally, regulation of gastrointestinal function occurs through the transmission of orexigenic and anorexigenic factors through the peripheral nervous system [16].

### 3.2. NNS Exposure and Endocrine Effect

Along with the other sweet-tasting compounds, NNS can activate the T1R2/T1R3 subunits, further inducing G protein stimulation and neuroendocrine and neurohormonal response [20]. Nevertheless, because NNS constitute a heterogeneous group of chemical compounds with different structures and metabolism, their further system transformation varies [21]. The site of NNS absorption is crucial in this regard. Only a part of them (saccharin, sucralose) is at least partially transported through the whole gastrointestinal tract, whereas some NNS (aspartame, acesulfame-K) are rapidly absorbed in the initial part of the gastrointestinal tract. Therefore, the stimulation of the sweet taste receptors in lower enteroendocrine cells does not occur when rapidly absorbed NNS are ingested [21]. It has been claimed that the consumption of NNS may induce the secretion of incretin hormones by intestinal enteroendocrine cells despite their low or zero energy value. However, this was observed only in vivo [22]. In contrast, there has been no increase in incretin secretion induced by NNS in humans in vivo [23,24]. Nevertheless, it should be emphasized that the lack of incretin secretion has only been demonstrated in studies in which NNS administration was not accompanied by food intake [21].

One of the mechanisms for postprandial blood glucose control and appetite regulation is the cephalic phase insulin response (CPIR). This response has a neurological basis and is independent of the increase in postprandial glucose level because an increase in insulin secretion occurs prior to nutrient absorption. CPIR may occur in response to food-related cues: sensory stimuli (sight, smell, taste, and texture of food) and other stimuli such as place and time of eating or even thinking about food. Oral stimulation is considered the key signal evoking this type of insulin response [25]. 

The NNS effects on CPIR are not completely elucidated yet. There is some evidence to suggest that oral exposure to some NNS (saccharin [26,27,28], sucralose [19]) can stimulate CPIR to the same extent as sucrose. On the other hand, alternative human studies have not demonstrated the ability of commonly used NNS (aspartame [29], acesulfame-K, cyclamate [30], sucralose [31]) to evoke CPIR [31,32]. The considerable discrepancy of results may be due to individual differences among subjects, including prior diet history and health status [25]. Future research should provide more insight into their impact on CPIR.

One of the hypothetical threats of chronic exposure to NNS is their negative impact on CPIR. It has been postulated that the consequence of consuming this type of food devoid of energy load may result in the disappearance of the cephalic phase insulin response and impair postprandial glycemic regulation [33]. However, this hypothesis is contradicted by a rodent study by Berthoud et al. [27], in which a 10-fold feeding of saccharin did not cause CPIR abolishment Nevertheless, little research has been conducted among regular NNS consumers [25]. It is suggested that other factors besides energy load may influence this response. Moreover, CPIR variability across subjects results from its multifactorial basis (e.g., BMI, dietary pattern). Finally, the evidence gathered so far does not fully support that CPIR is an important mechanism regulating feeding behavior in humans [23].

Some researchers believe that NNS can elicit CPIR leading to hypoglycemia and an increased desire to eat. However, the results of the studies conducted so far are not conclusive. In a study by Dhillon et al. [30], a statistically significant increase in insulin secretion after oral exposure to sucralose was observed in overweight and obese individuals. Similar results were observed by Just et al. [24] after saccharin oral administration to healthy participants. In contrast, the study conducted by Hartel et al. [26] did not confirm NNS (aspartame, acesulfame-K, and cyclamate) ability to increase insulin secretion because of CPIR induction. However, this effect was noted after the administration of saccharin. 

It is suggested that CPIR variability across subjects results from its multifactorial basis (e.g., BMI, dietary pattern). Finally, the evidence gathered so far do not fully support that CPIR is an important mechanism regulating feeding behavior in humans [25].

Taking into account an increased prevalence of disturbances of glucose homeostasis, such as insulin resistance and type2 diabetes mellitus, some investigations were conducted in an attempt to explain whether NNS contribute to the development of indicated disorders. There is some evidence that NNS may enhance insulin response after oral glucose administration. In a study by Pepino et al. [34], the insulin secretion was higher after sucralose than water in obese non-consumers of NNS. Sylvetsky et al. [35] reported an increase in insulin levels after consuming a diet-beverage containing aspartame, acesulfame-K, and sucralose in relation to water [35]. In contrast, studies conducted in healthy, normal-weight individuals found no effect of sucralose [24,36,37], aspartame [38,39], and saccharin [38] on insulin release in response to glucose. It has been suggested that the body weight of study participants may influence hormonal responses [35]. Obese people are known to be more predisposed to develop metabolic disorders, including insulin resistance and type 2 diabetes [40].

There are also reports that some NNS may affect insulin sensitivity. In a study by Romo-Romo et al. [41], a significant decrease in insulin sensitivity has been shown after moderate sucralose consumption in healthy individuals. The positive correlation between NNS consumption and impaired glucose tolerance has been also reported in mice in a Suez et al. study [42]. Moreover, the authors suggested that these changes may be mediated by NNS-induced microbiota dysbiosis. Nonetheless, there is no certainty whether results of this rodent study can be translated to humans. In contrast, no effect of NNS (beverage containing aspartame and acesulfame-K) on insulin sensitivity has been demonstrated in a trial conducted by Bonnet et al. [43] among nondiabetic adults. 

Based on the available evidence, it is difficult to determine conclusively whether NNS affect the risk of diabetes in humans [44]. Some studies report no effect on insulin and glucose. In turn, prospective cohort studies have shown a positive correlation between regular NNS use and type 2 diabetes risk. It is also worth mentioning that a possible explanation for this observation may be a reverse causality, as only baseline exposure is considered [21].

### 3.3. NNS and Appetite Control

The replacement of sugar by NNS as a weight control strategy is effective when reducing sugar intake entails a reduction in energy intake [6]. Nonetheless, there are doubts about the negative effects of NNS on appetite control, and therefore, risk of weight gain [45]. Some studies have aimed to assess whether the ingestion of NNS may contribute to compensatory increase in energy intake in response to greater appetite. 

In a longitudinal study lasting 18 months conducted by de Ruyter et al. [46], 203 children were assigned to two randomized assignment groups. Both groups were asked to replace their habitual daily sugar-sweetened drink with one can of the assigned drink each day. Nearly half of the participants received a sugar-sweetened beverage, whereas the remaining subjects received an NNS-containing beverage (sucralose, acesulfame-K). Every six months, participants recorded their subjective rating of hunger and satiety before pre- and post-beverage exposure. There was no significant difference between the two groups. Furthermore, after 18 months, all participants showed less desire to consume both types of beverages. On this basis, it was concluded that the use of NNS as sugar substitutes may support weight control due to the comparable effect on satiety. In another study conducted by Sorensen et al. [47], participants were divided into two groups to receive additional drinks and foods sweetened with sucrose or NNS. After ten weeks of intervention, it was observed that subjects receiving sugary products reported less satiety after lunch and dinner and were more likely to eat after these meals than participants who consumed NNS-sweetened foods. However, no significant differences were found between the two groups in subjective desires for sweetness. A similar level of satiety was reported by participants in the study by Anton et al. [48] after consuming NNS-sweetened foods (aspartame) compared to a meal containing sucrose. In both cases, no compensatory energy intake in subsequent meals was observed. A meta-analysis of clinical trials has shown that using NNS helps to reduce daily energy intake compared to the use of sugar. It has been observed in both normal-weight and overweight individuals [49].

In conclusion, the replacement of sucrose with NNS does not appear to contribute to short- and long-term compensatory energy intake. Even when this occurs, the compensation is not enough to result in weight gain or hinder weight loss [21]. However, in practice, many factors influence the dietary choices made each day. Therefore, the results of studies examining whether NNS contribute to compensatory energy intake may not translate to reality. It is emphasized that education and expanding nutritional awareness are key to successful weight management [6].

### 3.4. Neurobiological Aspect of Artificial Sweeteners Consumption 

In addition to homeostatic (hunger/satiety) brain regions, structures known as the hedonic brain regions are important regulators of eating behavior. This system promotes the search for and intake of food, which results in a strong feeling of pleasure. The secretion of neurotransmitters activates hedonic pathways by the hypothalamus, including dopamine and endogenous opioids, whose secretion increases after consuming food considered highly palatable. Hedonistic behaviors can be independent of the body’s energy needs, so consequently, overeating tasty, high-calorie foods can lead to excessive weight gain [27].

It is known that the consumption of sweet-tasting foods is a strong stimulus that activates the reward system. Nevertheless, the pleasure and satisfaction derived from their consumption vary depending on the NNS present. It has been suggested that the effect of NNS on the reward pathway differs from natural sweeteners. After eating NNS, it has been hypothesized that only partial activation of food reward pathways occurs, and this effect is explained by the separation of the actions of sweetness and energy value [50,51]. A reduction in reward response may increase appetite and thus food-seeking behavior. In turn, uncontrolled consumption of palatable sweet foods may result in weight gain [50].

In a study by Van Opstal et al. [52], the oral administration of glucose led to immediate activation of the reward system, whereas administration of sucralose resulted in only a small, short-term response comparable to that of water. In contrast, in another study, the ingestion of cocktails sweetened with sucralose or allulose, compared with cocktails containing glucose or fructose, induced a slight decrease in the activity of structures responsible for hedonic hunger. Nevertheless, studies investigating short-term effects have not shown an increased preference for sweet foods after frequent exposure to sweet stimuli [53]. No difference was noticed in sweet taste preferences due to increased consumption of sucrose or NNS sweetened beverages [54].

In recent years, the concept of “food addiction” has received increasing attention. It has been suggested that biochemical properties of certain foods (especially sugar and sweet-tasting foods) may result in the appearance of addiction-specific behaviors, including loss of control, withdrawal, food cravings, and binging, in susceptible individuals [55]. This view is quite controversial. In animal model studies, rats have been observed to exhibit behaviors associated with addiction when given intermittent access to sugar, but not with ad libitumaccess [56]. The results of human studies are not consistent, making it significantly more difficult to verify the concept of food (and sugar) addiction [57,58]. It is important to emphasize that the term “food addiction” is not currently classified as a mental disorder in the Diagnostic and Statistical Manual of Mental Disorders (DSM-V) [59]. More research is required to elucidate this phenomenon.

### 3.5. Changes in Sweetness Perception Induced by Energy Intake Reduction

Reduced caloric diet, together with physical activity and, in some cases, pharmacotherapy or surgical treatment, is one of the basic methods of overweight and obesity treatment [60].

**Figure 1:**
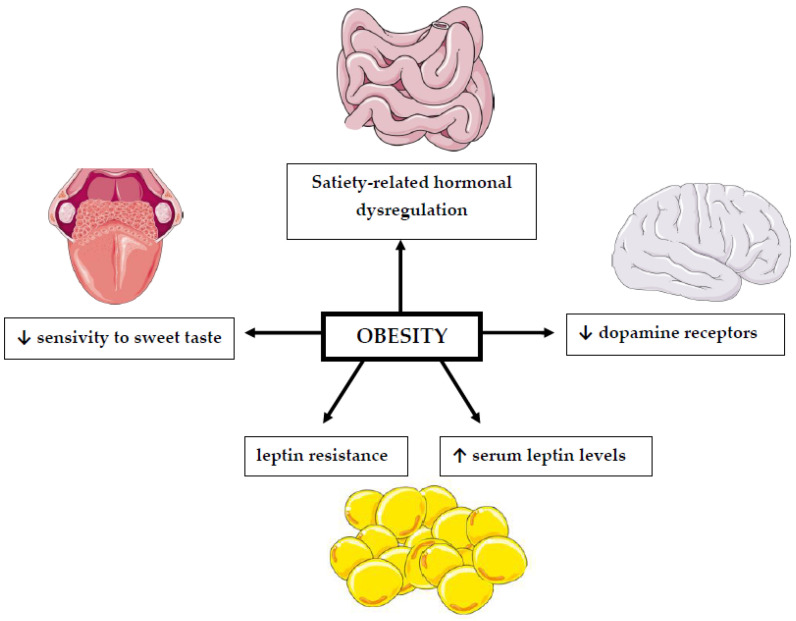
The relationship between obesity and impaired sweet taste perception. ↓: decrease; ↑: increase. This figure was made using Servier Medical Art collection (http://smart.servier.com/, accessed on 10 February 2022).

The association between BMI values and changes in the perception of sweet taste has been the subject of many studies for a long time [61,62] (Figure 1, Table 2). However, their results are inconclusive. Most of them suggest that people suffering from obesity have a reduced sensitivity to sweet taste [13,63]. However, this change is reversible and improves with weight loss [62]. This is an important issue because taste perception can influence consumers’ choices and the amount of consumed food [64]. The reason for the modification of taste perception in obesity, both sensory and hedonic, is due to changes in dopamine action. Overweight individuals have a reduced number of receptors for this neurotransmitter. Indeed, it has been established that increased body fat can cause a long-term reduction in dopamine receptors [65].

In a study conducted by Nishihara et al. [61], the influence of weight reduction, achieved by a calorie-restricted diet, on the changes in sweet taste perception was evaluated. In the initial part of the study, participants kept a food diary to determine their daily energy intake. However, in the intervention phase, the energy content of each participant’s diet was reduced by 500 kcal. The amount of simple sugars in the diet was also limited to less than 10%. The subjects were additionally advised to increase physical activity. Before the energy deficit diet, overweight subjects preferred a higher sucrose content more than the control group. However, after weight reduction, no significant difference was observed between the compared groups. No significant difference in sucrose detection threshold was detected between samples of subjects. The use of a reduction diet resulted in a decrease in the hedonic need to consume sweets in the obese group. The obtained changes in the preference of sweet taste and its palatability may result from the improvement of obesity status. Changes in the levels of hormones that regulate food intake, including leptin, may be a potential factor in taste modification [61]. The receptors for leptin are present in the brain reward system. Leptin’s ability to cross the blood–brain barrier and access central circuits is regulated by active transport. Obesity results in an elevated plasma level of leptin and resistance to this hormone. In obesity, transport mechanisms are saturable and become insensitive due to higher levels of leptin. Leptin receptors have been found in the ventral tegmental area (VTA). The VTA includes dopaminergic neurons, which innervate the nucleus accumbens (NAc) and create the mesolimbic dopamine system. Leptin acts on VTA and negatively regulates dopamine tone [66]. Since leptin affects the reward system in the brain, its elevated levels in obese individuals may account for their preference for sweet taste. It is also worth adding that areduction inmonosaccharides in the diet probably leads to adecrease in the hedonic response to the consumption of sweet food [61].

**Table 2:** Association of obesity–body mass index with sweet taste perception.

Study	Sample Group	Taste	Ratings	Results
Hardikar et al. [67]	23 people BMI > 30 kg/m^2^ 31 people BMI < 25 kg/m^2^ adults	sweet salty sour bitter	recognition threshold, hedonic response, intensity	People with a BMI > 30 are more sensitive to sweet taste and perceive it more intensely compared to lean people.
Skrandies et al. [68]	25 people BMI > 24.9 kg/m^2^ 36 people BMI 18.5–24.9 kg/m^2^ 5 people BMI < 18.5 kg/m^2^ adults	sweet salty sour bitter	recognition threshold	No effect of BMI on overall taste sensitivity has been demonstrated.
Vignini et al. [63]	30 people BMI < 24.9 kg/m^2^ 19 people BMI 25–29.9 kg/m^2^ 22 people BMI > 30 kg/m^2^ adults	sweet salty sour bitter	recognition threshold	Decrease in taste sensitivity as BMI increases.
Overberg et al. [69]	99 people > 97 centile 94 people < 90 centile children/teenagers age: 6–18	sweet sour salty bitter umami	taste sensitivity	Obese children are less sensitive to sweet taste and perceive it less intensely.

### 3.6. Weight-Loss Surgery and Sweet Taste Perception

The growing problem of obesity is associated with an increased number of bariatric surgeries performed. Surgical treatment of this disease is currently the most effective intervention for weight reduction and long-term maintenance of outcomes. Patients with BMI ≥ 40 kg/m^2^ or BMI ≥ 35 kg/m^2^ and comorbidities that can be improved by weight loss may be eligible for bariatric surgery [70].

In patients who have undergone bariatric surgery, changes are observed in sweet taste perception. However, it is not known whether this is due to the weight reduction achieved and the modification of eating habits or to the physiological changes caused by the surgical intervention [71]. The change in food preference may be due to the observed increased sensitivity to sweet taste and decreased sensation of pleasure after exposure to it. This may influence the type of foods chosen by individuals after bariatric surgery. Additionally, the avoidance of selected foods due to the feeling of discomfort after eating some of them, especially sweet and fatty foods [72].

A study conducted by Nielsen et al. [72] evaluated the sensory and hedonic modifications of taste perception after bariatric surgery. They examined whether there were differences between patients after the surgical treatment depending on the type of bariatric surgery performed-sleeve gastrectomy or Roux-en-Y Gastric Bypass (RYGB). RYGB patients were found to have increased sensitivity to sweet taste. However, it is worth mentioning that this has already been observed at the level of dietary intervention, so bariatric surgery is thought to be important but not the main contributor to this change. Weight reduction or reduction in carbohydrates and sweets in the diet may be the reason. The hedonic response to sweet taste has changed. In a group of people who underwent bariatric surgery, six months after the procedure, there was a decrease in the perception of pleasure after consuming sweet taste. Meanwhile, no such modification was observed in the group of individuals whose weight reduction was achieved through dietary intervention [71].

A study published in 2014 by Pepino et al. [73] controlled the change in taste perception and eating habits in women after bariatric surgery. Additionally, they examined whether there was a difference between RYGB and LAGB (laparoscopic adjustable gastric banding). Both types of surgeries were associated with changes in eating habits, and reductions in the desire to eat sweets and less frequent emotional eating were observed, among other things. Only patients after RYGB had a decreased hedonic response to sweet taste, regardless of the degree of weight reduction. However, there was no difference in sweet taste sensitivity between the two treatments. The study authors suggest that weight reduction and dietary modification, rather than surgical intervention, is the primary cause of the observed changes in participants’ eating behaviors. This is evidenced by the equal results obtained from the two different types of treatments [73].

Additionally, a study conducted by Altun et al. [74] evaluated sensitivity to different tastes in subjects scheduled for bariatric surgery. The test was conducted before the performed surgery, and one month and three months after SG. After the procedure was performed, a significantly increased sensitivity to sweet taste in particular was observed. The study’s authors point out that there is a small but positive correlation between the amount of excess body weight reduced and test results. They suggest that both weight reduction and hormonal changes observed after surgery affect taste sensitivity [74].

### 3.7. Influence of Changes in Body Composition on Sweet Taste Perception

There is a lack of research examining the association of achieved changes in body composition on sweet taste perception. However, a study by Lim et al. [75] examined whether there was a relationship between hedonic response to sweet taste and body composition, among other factors. Although individuals classified as liking sweet taste were observed to have a higher percentage of body fat compared to those in the non-sweet group, there was no association between sweet preference and body composition [75]. In contrast, in a study conducted by Iatridi et al. [76], as part of the taste test, the participants evaluated their enjoyment ofand sensitivity to sucrose. Depending on the hedonic response to sweet taste, three phenotypes were distinguished: sweet liker, sweet disliker, and optimal. Additionally, anthropometric measures were taken, including the assessment of total body fat and fat-free mass. The results suggest that the effect of sweetness preference on body composition changes with age. Those under 21 years of age who disliked sweet taste had higher body fat levels than older participants, whereas those who liked sweet taste at or above 21 years of age had a higher BMI and waist circumference and FFM (fat-free mass) than younger participants. The authors suggest that one of the reasons why sweet likers had higher BMI and waist circumference is lifestyle and increased exposure to obesogenic environment. Additionally, some behavioral characteristics may explain the anthropometric differences: sweet likers had higher sensitivity to rewarding stimuli than sweet dislikers. Additionally, the study authors note that there is a strong positive correlation between FFM and liking sweet taste. However, further research is needed [76].

In a study conducted by Umabiki et al. [62], changes in leptin levels and sweet taste detection threshold were evaluated after implementation of a 12-week dietary intervention and physical activity. Subjects showed reductions in body weight, BMI, waist circumference, percent body fat, and leptin concentrations and lower threshold for sweet taste [62]. Similar results were obtained in an animal model study. Shin et al. [77] examined the effects of obesity, weight loss, leptin, and genetic factors on liking and wanting to eat sweet and fatty foods. In the first part of the study, obesity was induced in rats through a high-fat or high-sugar diet. A change in taste preference was observed in rats with developed obesity. They preferred higher concentrations of sucrose compared to normal-weight individuals. In the next part of the study, a weight loss diet was used in obese rats. The energy content of the diet was restricted to 50–70% of daily requirements, with the goal of achieving a 20% weight loss in 3 weeks. After the dietary intervention, previously obese rats again preferred lower sucrose concentrations. The results suggest that the changes occurring in taste perception are obesity-induced and are reversible. According to the study authors, leptin is a potential cause of reduced sucrose sensitivity in obesity-induced individuals. Obese individuals had higher concentrations of leptin, compared to individuals of normal weight. To test whether this hormone can modulate sweet taste sensitivity, previously obese rats were given leptin at 1 mg/kg intraperitoneal (i.p.), and the control group was given saline. The reduced-weight rats, despite their previous preference for lower sucrose concentrations, again chose higher concentrations after leptin administration. Lowering leptin levels and improving leptin sensitivity through weight reduction can have a positive effect on sweet taste perception [77].

## 4. Sweeteners: Implications for Energy Intake and Body Weight Regulation

High-energy diets are one of the major contributors to overweight and obesity. Frequent consumption of foods high in sugar may cause excess of calories in the diet and contribute to excessive body weight [78]. This is important, especially in the context of the growing problem of obesity worldwide. Studies conducted by the OECD (Organisation for Economic Co-operation and Development) in 52 countries showed an increase in the percentage of obese people, which was 21% in 2010 and 24% in 2016 [79]. Therefore, the WHO recommends limiting the intake of simple sugars to 10% of daily energy intake [80].

Some observational studies and animal studies suggest a positive association between weight gain and NNS use [81]. 

In a systematic review with meta-analysis published in 2020, Laviada-Molina et al. [81] evaluated the effect of NNS on body weight and BMI [81]. In the study, in which energy-free foods such as water were the control sample, the use of NNS had no effect on weight change in contrast to studies that compared sugar with sweeteners. Replacing sucrose with NNS resulted in weight reduction. Additionally, it was noted that such results were most noticeable in those not on a calorie-restricted diet. The implementation of an energy-deficit diet translated into weight loss, making the use of NNS less relevant. It is also worth mentioning that in a reduction diet, simple sugars are usually restricted, whereas ad libitum feeding is usually associated with a higher energy intake and a higher content of simple sugars in the diet. For this reason, replacing sucrose with NNS may allow for a reduction in more simple sugars and energy. Some study authors have suggested that the effect of NNS on body weight is dependent on baseline BMI [46]. More significant effects of sugar substitution were observed in overweight or obese subjects, but not in normal-weight subjects. NNS themselves do not have weight-modifying properties. It is also worth noting that in this meta-analysis [81], NNS use was not shown to affect weight gain. In summary, the effects of NNS are most pronounced when they are used as a sugar substitute in overweight or obese individuals and in individuals not following an energy-deficit diet [81,82].

Similar results were obtained by Rogers et al. [83] in a systematic review with meta-analysis. Studies comparing the efficacy of sucrose and NNS found a greater reduction in body weight and BMI, and a more significant reduction in energy intake in the group using NNS. Additionally, weight loss was found to correlate positively with the percentage of sucrose replaced by sweeteners [83].

Additionally, a study conducted by Higgins et al. [84] evaluated the effects of sweeteners, including three NNS, aspartame, sucralose, saccharin, and sucrose, on body weight in overweight or obese subjects. After a 12-week intervention, an increase in body weight was observed in the group consuming sucrose or saccharin compared with those consuming other sweeteners. Reductions in body weight and energy intake were observed in those taking sucralose. No significant change in body weight was seen in the other groups. In addition, a difference was observed in the portion sizes consumed by the study participants. Compared to those taking NNS, the subjects using sucrose ate larger portions of food. On the other hand, smaller portions were consumed by subjects in the sucralose group, compared to subjects in the sucrose and aspartame groups. Additionally, participants taking sucrose were more likely to feel hungry. The study authors do not explain the mechanisms leading to the changes in body weight, but they point out that sucrose directly provides energy, unlike NNS, which “displace other energy sources from the diet”. They observed no link between sucrose and energy intake despite increases in appetite and hunger. A potential cause of the weight gain could be a slowing of metabolism and increased energy acquisition by the gut microbiota. However, confirmation of this hypothesis would require further research in this direction [84].

It is also worth mentioning that people trying to reduce energy intake and body weight are much more likely than those not on a reduction diet to choose reduced-calorie products with added sweeteners [85]. In this regard, it should be noted that foods with added NNS could have important psychological significance. By helping to satisfy the appetite for sweet taste, it may, at the same time, contribute to the reduction or elimination of snacking on high-energy foods [86].

This intervention appears to have a beneficial effect on body weight and glycemic levels. Additionally, it was pointed out that consuming unhealthy foods with added sweeteners does not make them healthy, but they are better alternatives. A similar opinion was issued by the British Dietetic Association (BDA), confirming the potentially positive effect of this substitution on body weight regulation [86].

## 5. Conclusions

Since food preferences and dietary habits are set from a young age, there is a need for nutrition education intervention on reducing consumption of sugar-rich products. Moreover, liability is assumed by the food industry to make every effort to reduce the sugar content in packed foods. Amid rising obesity rates, NNS have been proposed as a potentially useful tool for weight management. Based on studies conducted so far, it can be assumed that replacing sucrose-sweetened foods with products containing NNS could be an effective nutritional intervention to reduce excessive body weight and prevent overweight and obesity. The beneficial effect of NNS use on body weight is primarily due to a reduction in dietary energy intake and is therefore dependent on energy deficit. Nor has NNS consumption been proven to provide a direct stimulus for increased food intake; nevertheless, deliberate compensations may alter or even reverse beneficial effect. On the contrary, the dietary substitution of simple sugars accompanied by weight loss may contribute to a reduced preference for sweet taste due to changes in neurohormonal and neurobiological perception of taste. However, many questions about the health effects of NNS have yet to be resolved. More well-designed studies are needed to understand the long-term effects of NNS on weight status, appetite control, and their impact on sweetness perception.

## Figures and Tables

**Table 1:** The comparison of non-nutritive sweeteners.

Sweetener	No. E	Sweetness Compared to Sucrose	Characteristic	Energy	ADI (mg/kg Body Weight/Day)	Other
Aspartame	E951	200×	- unstable at high temperatures (cannot be used for cooking/baking) - degrades over time in beverages - slightly soluble in water, solubility increases at higher temperatures and at acidic or basic pH	4 kcal/g	50	- must not be used by people suffering from phenylketonuria (PKU) - does not cause tooth decay - despite numerous controversies recognized as safe for use in doses not exceeding 40 mg/kg body weight/day - in particular added to carbonated soft drinks type 0/no sugar added, but also to the manufacture of medicines
Acesulfame Potassium (Ace-K)	E950	200×	- stable at high temperatures; therefore, can be used for baking and cooking - soluble in water	is not metabolized in the body and therefore does not provide energy	15	- due to its ability to leave a bitter aftertaste, it is most often used with other sweeteners - mostly added to sodas and chewing gums, but also to salty and sweet snacks
Saccharin	E954	200–700×	- stable at high temperatures	is not metabolized in the body and therefore does not provide energy	15	- because of its ability to leave a bitter aftertaste, it is most often used with other sweeteners - mostly added to soft drinks and juices, jams, chewing gum, dairy products, and cookies
Sucralose	E955	600×	- stable at high temperatures - soluble in water - stable at various pH values	is not metabolized in the body and therefore does not provide energy	5	- sucrose derivative - added to drinks and juices, pharmaceutical products
Neotame	E961	7000–13,000×	- freely soluble in alcohol, slightly soluble in water - odorless, white powder	does not provide energy	0–2	- rapidly metabolized in the body but is completely eliminated - used in the production of cookies, yoghurt, carbonated drinks, chewing gum

## Data Availability

Not applicable.
